# RSSI-WSDE: Wireless Sensing of Dynamic Events Based on RSSI

**DOI:** 10.3390/s24154952

**Published:** 2024-07-31

**Authors:** Xiaoping Tian, Song Wu, Xiaoyan Zhang, Lei Du, Sencao Fan

**Affiliations:** College of Information Engineering, Beijing Institute of Petrochemical Technology, Beijing 102617, China; 2022540068@bipt.edu.cn (S.W.); 2023520352@bipt.edu.cn (X.Z.); 2021540034@bipt.edu.cn (L.D.); 2023540099@bipt.edu.cn (S.F.)

**Keywords:** dynamic event sensing, time series analysis, received signal strength indication

## Abstract

Wireless sensing is a crucial technology for building smart cities, playing a vital role in applications such as human monitoring, route planning, and traffic management. Analyzing the data provided by wireless sensing enables the formulation of more scientific decisions. The wireless sensing of dynamic events is a significant branch of wireless sensing. Sensing the specific times and durations of dynamic events is a challenging problem due to the dynamic event information is concealed within static environments. To effectively sense the relevant information of event occurrence, we propose a wireless sensing method for dynamic events based on RSSI, named RSSI-WSDE. RSSI-WSDE utilizes variable-length sliding windows and statistical methods to process original RSSI time series, amplifying the differences between dynamic events and static environments. Subsequently, z-score normalization is employed to enhance the comparability of the sensing effects for different dynamic events. Furthermore, by setting the adaptive threshold, the occurrence of dynamic event is sensed and the relevant information is marked on the original RSSI time series. In this study, the sensing performance of RSSI-WSDE was tested in indoor corridors and outdoor urban road environments. The wireless sensing of dynamic events, including walking, running, cycling, and driving, was conducted. The experimental results demonstrate that RSSI-WSDE can accurately sense the occurrence of dynamic events, marking the specific time and duration with millisecond-level precision. Moreover, RSSI-WSDE exhibits robust performance in wireless sensing of dynamic events in both indoor and outdoor environments.

## 1. Introduction

In the context of building smart cities [1], wireless sensing technology has attracted significant attention in the research community due to its extensive integration in sensor networks and monitoring systems. Furthermore, as a critical enabling technology of the internet of things (IoT), wireless sensing technology [2] demonstrates strong capabilities in independent sensing, computing, and communication. Based on IoT and cloud computing, Dong et al. utilized microseismic monitoring, image recognition technology, artificial intelligence training, and smart sensors to locate the autonomous driverless vehicles in mining scenarios. This extends the research scope of autonomous driving technology in the development of smart cities [3]. Moreover, by integrating sensor networks, wireless sensing technology can accomplish a series of complex tasks, including data collection, data processing, data fusion [4,5], distributed computing [6], and the collaborative work among multiple nodes in the network. Therefore, the application of wireless sensing technology in the construction of smart cities is becoming increasingly crucial, providing new perspectives and solutions for urban management and services.

Event sensing, as one of the core applications of wireless sensing systems [7], is used to capture and analyze events occurring in specific environments. This technology has been widely applied in various fields of production and daily life, such as human body monitoring [8], smart agriculture [9], and intelligent transportation [10]. In the field of human body monitoring, event sensing technology enables fall monitoring for elderly people and patients [11], self-powered monitoring of human respiration [12], and behavior detection of intrusions into buildings [13]. In smart agriculture, this technology not only facilitates the positioning of farm machinery in the field [14], but also monitors the growth of crops in the field [15], significantly promoting the intelligence of agricultural production. Applications in smart transportation include the dynamic monitoring of reckless driving [16] and the monitoring of traffic conditions [17], effectively reducing accident rates and optimizing road traffic flow management. These diverse application scenarios not only highlight the widespread applicability of event sensing in practical setting, but also promote in-depth research into various types of event sensing tasks. Therefore, researchers are dedicated to using low-cost, low-power, and lightweight sensors to construct wireless sensing systems. Their goal is to efficiently sense specific events and provide services to users.

The majority of wireless sensing devices come equipped with integrated RSSI circuits and modules, obviating the necessity for additional devices. These reduce the hardware cost and power consumption of the wireless sensing system. Hence, RSSI is widely used in the field of wireless sensing [18,19]. When dynamic events affect the radio frequency signal strength of wireless sensing devices, it leads to a more dispersed histogram distribution of RSSI, which statistically manifests as a higher standard deviation [20]. Therefore, it is necessary to consider the fluctuation in signal strength caused by non-event-triggered interference data, which may lead to erroneous sensing results. Furthermore, using a time window to process the RSSI time series is a critical method for event sensing [21]. It is necessary to set the appropriate window length according to the different sensing tasks to enhance efficiency and accuracy. In particular, when executing wireless sensing tasks, it is imperative to fully consider the issue of data transmission delay. Ren et al. proposed a time-balanced scheduling data collection scheme, which minimizes data transmission delay issues through seamless data relay and joint scheduling methods [22].

It is particularly crucial to design a lightweight wireless sensing method that performs reliably across various types of events in indoor and outdoor settings. Therefore, we propose a wireless sensing method for dynamic events based on RSSI (RSSI-WSDE). This method integrates variable-length sliding window and statistical methods to process the RSSI time series. It effectively reduces the interference from the static environment on dynamic event sensing. Subsequently, by setting adaptive thresholds, occurrences of dynamic events are sensed wirelessly. Furthermore, the specific times and durations of events are accurately marked. In indoor corridor environments, RSSI-WSDE concentrates on the sensing issues within the human domain, primarily addressing the sensing tasks for walking dynamic events. In outdoor road settings, while continuing to focus on the human domain, RSSI-WSDE also integrates the perception of vehicular domains, principally addressing sensing tasks associated with walking, running, cycling, and driving dynamic events.

Applying RSSI-WSDE to hardware devices enables the establishment of the dynamic event wireless sensing system. The system is intended to be applied in scenarios primarily targeting late-night periods with low traffic flow, sensing the motion trajectories of target objects. Its primary objective is to assist public security departments in accurately determining the movements of suspects in complex nighttime environments, where surveillance video footage may be unclear or blind spots exist. Specifically, the system employs ZigBee devices operating in the 2.4 GHz frequency band. ZigBee devices are characterized by their low power, low cost, and high flexibility, enabling rapid self-organization of networks to ensure stable and reliable data transmission. Moreover, due to their long communication range and wide coverage, ZigBee devices are capable of performing RSSI time series data collection tasks in both indoor and outdoor environments.

The main contributions of this study can be summarized as follows:

(1) A wireless sensing system of dynamic event is developed, consisting of multiple collection units, each equipped with a set of transmitter and receiver units. The transmitter units are connected to the host computer via data transmission line to display the RSSI waveform changes and sensing outcomes caused by dynamic events.
(2) An efficient wireless sensing algorithm of dynamic event is designed. It combines a variable-length average sliding window and variance sliding window to obtain the sliding average sequence and the sliding variance sequence. Subsequently, the variance amplification sequence is generated via amplification processing. Furthermore, by employing the sliding window technique along with a long-term data processing strategy, the algorithm calculates the z-score to obtain the sequence Z that reflects the occurrence of dynamic events. Finally, by setting adaptive thresholds, occurrences of dynamic events are sensed, and the information of event occurrence specific time and duration is marked.
(3) An adaptive threshold method for RSSI-WSDE is proposed. This method effectively distinguishes static environments from dynamic events in the complete RSSI time series. Furthermore, it accurately captures the specific times and durations of dynamic events.
(4) Dynamic event data collection experiments were conducted in indoor and outdoor environments to verify the effectiveness and practicality of RSSI-WSDE. Indoor experiments primarily focused on collecting RSSI data for walking, while outdoor experiments included collection of RSSI data for walking, running, cycling, and driving. The experimental results demonstrate that the RSSI-WSDE proposed in this study effectively senses the occurrence of dynamic events.

The remainder of this paper is organized as follows. Section 2 reviews and categorizes research works on event sensing. Section 3 introduces the network architecture and research method of RSSI-WSDE. Section 4 presents the description of the indoor and outdoor data collection experiments and the analysis of the experimental results. Finally, the conclusion is given in Section 5.

## 2. Related Works

In this section, we review and categorize the extensive existing research works on RSSI-based event sensing.

Event sensing can be classified into static and dynamic categories based on the state of the sensed object. Static event sensing primarily focuses on detecting the relative stationary state of the sensed object to capture the static characteristics of the event. Li et al. proposed a parking occupancy detection method based on the CC1101 wireless communication chip [23]. This method measures the impact of vehicles on the received signal strength indicator (RSSI) of parking spaces and sets appropriate thresholds, effectively detecting the occupancy status of parking spaces. Additionally, Wounchoum et al. explored the impact of the human body on RSSI at different positions in single line-of-sight and multiple line-of-sight wireless links. This research provides a theoretical and practical basis for device-free human sensing systems based on RSSI [24]. On the other hand, the dynamic event refers to an event that involves displacement, movement, and changes within a specific time and space, characterized by a real-time and continuous nature. Thus, dynamic event sensing focuses on these moving characteristics, aiming to track and capture changes in event states, thereby supporting subsequent data analysis, prediction, and processing tasks. Based on the environment where the event occurs, dynamic event sensing can be further divided into indoor and outdoor dynamic event sensing.

The current research on indoor dynamic event wireless sensing primarily focuses on the sensing of human activity events. Mrazovac et al. proposed a smart residential energy system based on sensing a human RSSI. This system detects human activities and enables intelligent control of power output and light switches [25]. Additionally, Booranawong et al. developed a device-free human sensing and tracking system using RSSI. By filtering the RSSI and employing adaptive thresholds and region selection functions, the actual area of human activity was accurately sensed, thus enhancing sensing accuracy [26]. However, the threshold setting mechanism of this system has limitations when dealing with more complex human activity patterns. Therefore, Booranawong et al. further proposed an adaptive RSSI filtering method that automatically filters RSSI inputs, exhibiting strong variation levels [27]. Furthermore, Lin et al. proposed a method to sense human activities by analyzing the wireless irregularities caused by the human movement across the signal transmission path. This approach maps the RSSI fluctuation to a normal distribution and uses the probability that the fluctuation falling within the [−1, 1] range as the threshold to determine whether someone is moving along the signal transmission path [28]. Additionally, Lin et al. proposed a method for sensor network formation capable of sensing RSSI fluctuation patterns related to specific objects. By utilizing the RSSI between transceivers in the network, the number of people passing and crossing the signal transmission path can be sensed and counted [29].

The current research in outdoor dynamic event wireless sensing primarily focuses on the sensing of vehicle movement events. Intelligent transportation systems represent the forefront of global transportation research, with cooperative vehicle infrastructure systems as a key component. These systems utilize wireless communication and sensing technologies to enable intelligent coordination and cooperation in vehicle-to-vehicle (V2V) and vehicle-to-infrastructure interactions. By leveraging these technologies, it optimizes the utilization of road network resources, alleviates traffic congestion, and enhances the overall operation of the road network [30,31]. Zhang et al. proposed a method combining Exponential Bloom Filter sketches and exponential histograms to effectively sense anomalies such as vehicle overloads in traffic flows by querying data streams within a sliding window [32]. Additionally, Rai et al. proposed an overtaking assistance system based on RSSI to prevent traffic accidents in V2V environments [33]. This system employs directional antennas and custom analog readout units to reflect the mapping relationship between vehicle speeds and RSSI variations at signal reception points. Duan et al. presented a traffic state sensing method based on vehicle-to-everything wireless signals [17]. This method analyzes the propagation characteristics of wireless signals at intersections. Specifically, it calibrates model parameters through a simulation platform that collects the RSSI between vehicle nodes and roadside units. This process ultimately achieves measurements of traffic flow parameters such as flow speed, flow rate, flow density at intersection entry lanes, and queue lengths in dedicated left-turn lanes.

## 3. Architecture and Research Methods

The main notations used in the following sections are summarized in Table 1.

### 3.1. Problem Definition

This study discusses the key challenges faced when using RSSI-WSDE. The primary challenge is how to enhance the distinction between dynamic events and static environments. By significantly amplifying the differences, the completeness and reliability of dynamic event sensing are improved. It ensures that the sensed RSSI clearly reflects the characteristics of dynamic events.

Secondly, for the dynamic event RSSI data obtained through wireless sensing, setting an adaptive threshold and marking the specific time and duration are technical challenges in implementing dynamic event wireless sensing. By marking the specific times of the dynamic events, the sequence of different events can be determined, providing a basis for tracking and reconstructing the motion trajectory of the dynamic event execution. Additionally, the durations of the dynamic events can serve as characteristic data for event type classification. Specifically, this study primarily illustrates how RSSI can be utilized to sense the occurrence of dynamic events and mark their specific times and durations in the time domain.

Finally, this study examines how variations in device sampling frequency and sliding window length affect the effectiveness of the dynamic event wireless sensing. Changes in the sampling frequency are closely related to the accuracy of wireless sensing. Appropriate sampling frequencies should be configured for devices according to different event scenarios to perform sensing tasks. Since devices with different sampling frequencies collect RSSI time series data of varying magnitudes, the corresponding sliding window length should be adjusted during data processing based on the sampling frequency. The length of the sliding window determines the granularity of dynamic event wireless sensing. Setting the window too short increases the computational complexity and amplifies the noise interference, while setting the window too long extends the data processing span, obscuring detailed information in the data.

### 3.2. Network Architecture

**Figure 1:**
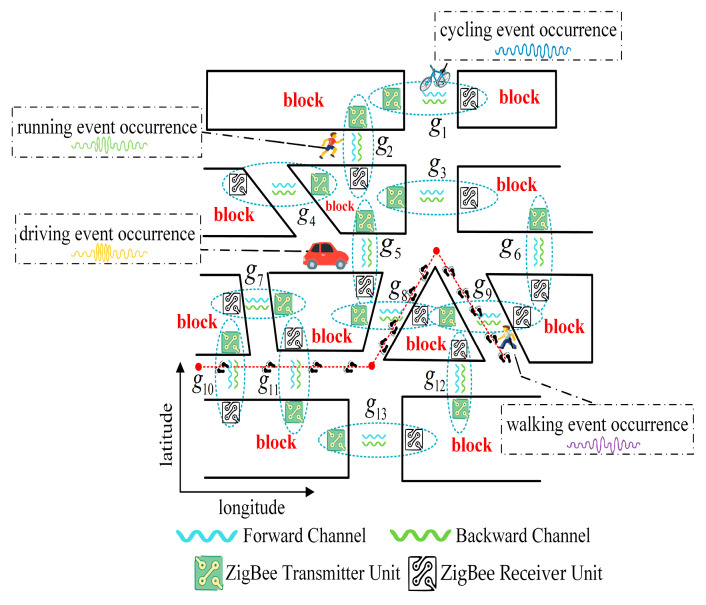
Schematic diagram of dynamic event wireless sensing network.

A dynamic event wireless sensing system composed of multiple collection unit device groups is considered in this study, and is represented by the set $G≜\left\{g_{1},g_{2},\ldots ,g_{i}\right\}$, as shown in Figure 1.

Figure 1 illustrates the schematic of the dynamic event wireless sensing network. The deployment of the system takes into account geographical location information and focuses on deploying it in critical connectivity zones within urban blocks. This measure optimally utilizes the spatial structure of roadways, enhancing the effectiveness and rationality of the device layout. Therefore, the system is deployed at traffic nodes such as intersections, three-way intersections, and other multi-junction intersections. As shown in Figure 1, five collection unit device groups ($g_{3}$, $g_{5}$, $g_{6}$, $g_{8}$, $g_{9}$) are deployed at the multi-intersection junction to monitor the occurrence of dynamic events in different directions. Additionally, for any collection unit device group $g_{i}\in G$, it is responsible for collecting the RSSI time series data of various dynamic events including walking, running, cycling, and driving. As the event execution objects move through different sensing areas, device groups can sense the occurrence of dynamic events and plot the corresponding RSSI waveforms.

To analyze the variations in the data fluctuation and distribution of dynamic events across different collection channels, the collection channels are divided into forward and backward channels based on the direction of data transmission. By processing dual-channel dynamic event RSSI time series data, the specific times and durations of various events are obtained. Subsequently, the time domain sensing information will be uploaded to the database. This provides data support and empirical evidence for reconstructing the motion trajectories of the dynamic event execution objects. Taking the motion trajectory marked with the red dashed line in Figure 1 as an example, the system infers the type of the event based on its duration and reconstructs the motion trajectory of the walking event execution object using the sequence of specific times.

For any collection unit device group $g_{i}\in G$, it consists of a set of ZigBee transmitter and receiver units. In order to prevent inter-group channel interference, each collection unit device group is assigned a different personal area network identifier (PANID). This measure not only improves the security and stability of the network, but also effectively avoids interference from other wireless devices operating in the 2.4 GHz band. Because PANID is able to allocate independent workspaces for different collection unit device groups within the fixed 2.4 GHz band, multiple groups of devices can coexist without interference in the same physical area. Only ZigBee devices with the same PANID are able to establish communication connections. Furthermore, ZigBee operates based on the IEEE 802.15.4 standard, employing frequency hopping strategies to mitigate interference from other wireless devices. By dividing the 2.4 GHz band into 16 different channels, ZigBee effectively reduces the likelihood of channel overlap and interference, ensuring the reliability of data transmission.

### 3.3. RSSI-WSDE

In order to enhance the comparability of the RSSI time series of dynamic events in different collection channels, dual-channel data collection is employed, represented by channel X and channel Y. In this setup, the RSSI time series processed by channel X is denoted as $\left\{x_{1},x_{2},x_{3},\ldots \right\}$, and the RSSI time series processed by channel Y is denoted as $\left\{y_{1},y_{2},y_{3},\ldots \right\}$, with n representing the data points contained in each channel.

#### 3.3.1. Average Sliding Window

**Figure 2:**
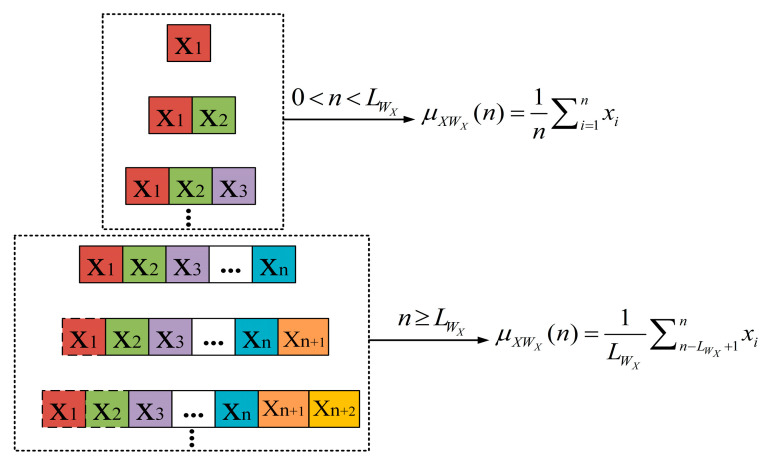
Schematic diagram of the sliding average sequence calculation in channel X.

In order to smooth out random fluctuations and noise in the original RSSI time series, making the main trend and distribution characteristics more apparent, the average sliding window method is employed to calculate the sliding average sequence of channel X and channel Y. In each channel, the sliding window is represented by $W_{X}$ and $W_{Y}$, respectively. The lengths of the sliding windows are represented by $L_{W_{X}}$ and $L_{W_{Y}}$, respectively. The schematic of the sliding average sequence calculation in channel X is shown in Figure 2. When the cumulative data points have not yet reached the sliding window length, the length of the sliding window is updated using the current number of data points to avoid computation delays caused by insufficient data within the window. Once the number of data points reaches the length of the sliding window, a fixed window length is used to maintain the consistency in data processing.

Each time the window slides by one data point, the RSSI data in the channel are divided into different sliding windows. The processing method depends on the relationship between the number of data points and the length of the sliding window. Specifically, the variable-length sliding window adapts well to changes in data length within the RSSI time series, allowing for flexible extraction of feature variations across different data segments. Utilizing smaller sliding windows enhances processing efficiency when the RSSI time series contains fewer data points. As the data in the RSSI time series continue to accumulate, adjusting the window length accurately captures distributional features within the data segments. This approach not only improves the efficiency and accuracy of data processing, but also enables flexible application across various scenarios, ensuring a timely response to data changes and precise analysis.

The sliding average sequences of channels X and Y are denoted as $\left\{\mu_{XW_{X}}(1),\mu_{XW_{X}}(2),\mu_{XW_{X}}(3),...\right\}$ and $\left\{\mu_{YW_{Y}}(1),\mu_{YW_{Y}}(2),\mu_{YW_{Y}}(3),...\right\}$, respectively.

In channel X, the original data sample is represented by $x_{i}$, and the calculation method of the parameter value $\mu_{XW_{X}}(n)$ of the sliding average sequence in channel X is as follows:

(1) $$\mu_{XW_{X}}(n)=\left\{\begin{array}{c} \frac{1}{n}\sum_{i=1}^{n}x_{i},0<n<L_{W_{X}}\\ \frac{1}{L_{W_{X}}}\sum_{n-L_{W_{X}}+1}^{n}x_{i},n\geq L_{W_{X}} \end{array}\right.$$

In channel Y, the original data sample is represented by $y_{i}$, and the calculation method of the parameter value $\mu_{YW_{Y}}(n)$ of the sliding average sequence in channel Y is as follows:

(2) $$\mu_{YW_{Y}}(n)=\left\{\begin{array}{c} \frac{1}{n}\sum_{i=1}^{n}y_{i},0<n<L_{W_{Y}}\\ \frac{1}{L_{W_{Y}}}\sum_{n-L_{W_{Y}}+1}^{n}y_{i},n\geq L_{W_{Y}} \end{array}\right.$$

To analyze the difference between data in channel X and channel Y, channel Y is used as the reference channel. The difference between the corresponding data points of the two channels is calculated to obtain the difference channel, denoted channel D. The data parameter $d_{i}$ is represented by $x_{i}-y_{i}$ in channel D. Furthermore, the sliding average sequence is denoted $\left\{\mu_{DW_{D}}(1),\mu_{DW_{D}}(2),\mu_{DW_{D}}(3),...\right\}$. The calculation method of the parameter value $\mu_{DW_{D}}(n)$ of the sliding average sequence is as follows:

(3) $$\mu_{DW_{D}}(n)=\left\{\begin{array}{c} \frac{1}{n}\sum_{i=1}^{n}\left(x_{i}-y_{i}\right),0<n<L_{W_{D}}\\ \frac{1}{L_{W_{D}}}\sum_{n-L_{W_{D}}+1}^{n}\left(x_{i}-y_{i}\right),n\geq L_{W_{D}} \end{array}\right.$$

where n represents the number of data points,$W_{D}$ represents the sliding window, and $L_{W_{D}}$ represents the length of the sliding window.

Referring to the calculation methods of the sliding average sequence parameter value $\mu_{XW_{X}}(n)$ of channel X and $\mu_{YW_{Y}}(n)$ of channel Y, the RSSI data in channels X and Y are processed using the sliding window $W_{D}$. The obtained sliding average sequence parameter values are represented as $\mu_{XW_{D}}(n)$ and $\mu_{YW_{D}}(n)$, respectively. The sliding average sequence parameter value $\mu_{DW_{D}}(n)$ of channel D is as follows:

(4) $$\mu_{DW_{D}}(n)=\mu_{XW_{D}}(n)-\mu_{YW_{D}}(n)$$

#### 3.3.2. Variance Sliding Window

As the window slides, new data are added while old data are removed; thus, the variance within the window is continuously updated. This dynamic updating is particularly effective for monitoring the dispersion and trend changes in RSSI time series data. Using the data processing method of the variance sliding window, the sliding variance sequences of channels X and Y are calculated, denoted $\left\{\sigma_{XW_{X}}^{2}(2),\sigma_{XW_{X}}^{2}(3),\sigma_{XW_{X}}^{2}(4),...\right\}$ and $\left\{\sigma_{YW_{Y}}^{2}(2),\sigma_{YW_{Y}}^{2}(3),\sigma_{YW_{Y}}^{2}(4),...\right\}$, respectively.

In channel X, the parameter value of the sliding variance sequence is represented by $\sigma_{XW_{X}}^{2}(n)$. Combined with the calculation result of the sliding average sequence parameter value $\mu_{XW_{X}}(n)$, the calculation method of $\sigma_{XW_{X}}^{2}(n)$ is as follows:

(5) $$\sigma_{XW_{X}}^{2}(n)=\left\{\begin{array}{l} \frac{1}{n}\sum_{i=1}^{n}{\left(x_{i}-\mu_{XW_{X}}(n)\right)}^{2},1<n<L_{W_{X}}\\ \frac{1}{L_{W_{X}}}\sum_{i=n-L_{W_{X}}+1}^{n}{\left(x_{i}-\mu_{XW_{X}}(n)\right)}^{2},n\geq L_{W_{X}} \end{array}\right.$$

In channel Y, the parameter value of the sliding variance sequence is represented by $\sigma_{YW_{Y}}^{2}(n)$. Combined with the calculation result of the sliding average sequence parameter value $\mu_{YW_{Y}}(n)$, the calculation method of $\sigma_{YW_{Y}}^{2}(n)$ is as follows:

(6) $$\sigma_{YW_{Y}}^{2}(n)=\left\{\begin{array}{l} \frac{1}{n}\sum_{i=1}^{n}{\left(y_{i}-\mu_{YW_{Y}}(n)\right)}^{2},1<n<L_{W_{Y}}\\ \frac{1}{L_{W_{Y}}}\sum_{i=n-L_{W_{Y}}+1}^{n}{\left(y_{i}-\mu_{YW_{Y}}(n)\right)}^{2},n\geq L_{W_{Y}} \end{array}\right.$$

To analyze the differences in the dispersion of data between channels X and Y, the sliding variance sequence of channel D is obtained using the variance sliding window. This sliding variance sequence is denoted by $\left\{\sigma_{DW_{D}}^{2}(2),\sigma_{DW_{D}}^{2}(3),\sigma_{DW_{D}}^{2}(4),...\right\}$.

In channel D, the parameter value of the sliding variance sequence is represented by $\sigma_{DW_{D}}^{2}(n)$. Combined with the calculation result of the sliding average sequence parameter value $\mu_{DW_{D}}(n)$, the calculation method of $\sigma_{DW_{D}}^{2}(n)$ is as follows:

(7) $$\sigma_{DW_{D}}^{2}(n)=\frac{1}{L_{W_{D}}}\sum_{i=n-L_{W_{D}}+1}^{n}{\left(d_{i}-\mu_{DW_{D}}(n)\right)}^{2}$$

When the current number of data points in channel D reaches the sliding window length, the calculation method of $\sigma_{DW_{D}}^{2}(n)$ is as follows:

(8) $$\begin{array}{l} \sigma_{DW_{D}}^{2}(n)=\frac{1}{L_{W_{D}}}{\sum_{i=n-L_{W_{D}}+1}^{n}\left(x_{i}-\mu_{XW_{D}}(n)\right)}^{2}\\ +\frac{1}{L_{W_{D}}}{\sum_{i=n-L_{W_{D}}+1}^{n}\left(y_{i}-\mu_{YW_{D}}(n)\right)}^{2}\\ -\frac{2}{L_{W_{D}}}\sum_{i=n-L_{W_{D}}+1}^{n}\left(x_{i}y_{i}-x_{i}\mu_{YW_{D}}(n)-y_{i}\mu_{XW_{D}}(n)+\mu_{YW_{D}}(n)\mu_{XW_{D}}(n)\right) \end{array}$$

When the current number of data points is less than the sliding window length, the calculation method of $\sigma_{DW_{D}}^{2}(n)$ is as follows:

(9) $$\begin{array}{l} \sigma_{DW_{D}}^{2}(n)=\frac{1}{n}{\sum_{i=1}^{n}\left(x_{i}-\mu_{XW_{D}}(n)\right)}^{2}\\ +\frac{1}{n}{\sum_{i=1}^{n}\left(y_{i}-\mu_{YW_{D}}(n)\right)}^{2}\\ -\frac{2}{n}\sum_{i=1}^{n}\left(x_{i}y_{i}-x_{i}\mu_{YW_{D}}(n)-y_{i}\mu_{XW_{D}}(n)+\mu_{YW_{D}}(n)\mu_{XW_{D}}(n)\right) \end{array}$$

Referencing Equation (5) for calculating the sliding variance sequence parameter value $\sigma_{XW_{X}}^{2}(n)$ of channel X and Equation (6) for the sliding variance sequence parameter value $\sigma_{YW_{Y}}^{2}(n)$ of channel Y, and using the sliding window $W_{D}$ to process data in channels X and Y, the obtained sliding variance sequence parameters are represented by $\sigma_{XW_{D}}^{2}(n)$ and $\sigma_{YW_{D}}^{2}(n)$, respectively. The sliding variance sequence parameter value $\sigma_{DW_{D}}^{2}(n)$ of channel D is as follows:

(10) $$\sigma_{DW_{D}}^{2}(n)=\sigma_{XW_{D}}^{2}(n)+\sigma_{YW_{D}}^{2}(n)-2cov_{W_{D}}(X,Y),n>1$$

where $cov_{W_{D}}(X,Y)$ represents the covariance of the data in channels X and Y processed by sliding window $W_{D}$.

In order to effectively enhance data utilization and analytical precision while reducing the loss of dynamic event information and capturing richer context data features, this study employs the overlapping sliding window method to calculate the sliding average sequence and sliding variance sequence of each channel. Through this method, individual data points are repeatedly utilized across multiple windows, enhancing the analysis of the associations between adjacent data points, thus more accurately reflecting the dynamic changes of the data.

#### 3.3.3. Variance Amplification Sequence

**Figure 3:**
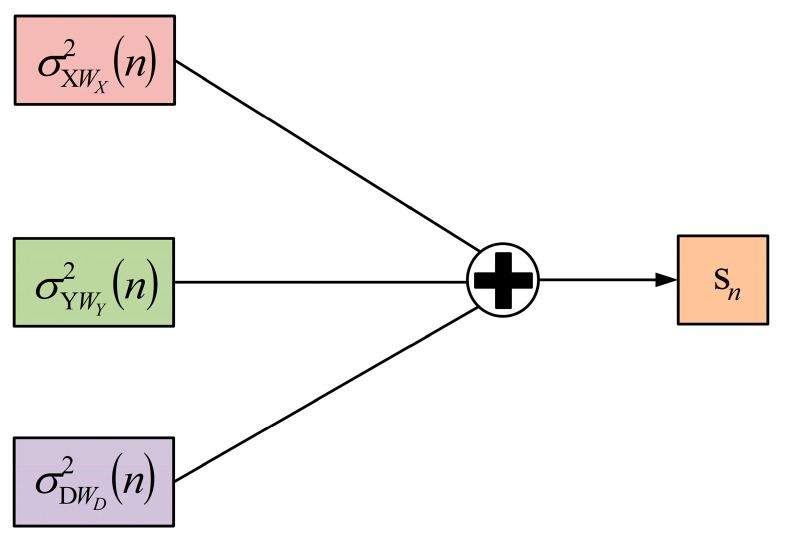
The three-channel sliding variance sequence amplification model.

In order to significantly enhance the difference between dynamic events and static environmental data, the sliding variance sequences of channels X, Y, and D have been amplified to obtain the variance amplification sequence S. The three-channel sliding variance sequence amplification model is shown in Figure 3.

In the three-channel sliding variance sequence amplification model, the parameters of the sliding variance sequences from three channels are sequentially added to obtain the variance amplification sequence, denoted sequence S, which can be expanded as $\left\{s_{2},s_{3},s_{4},\ldots \right\}$. The calculation method of data sample $s_{n}$ in sequence S is as follows:

(11) $$s_{n}=\sigma_{XW_{X}}^{2}(n)+\sigma_{YW_{Y}}^{2}(n)+\sigma_{DW_{D}}^{2}(n),n>1$$

In channels X, Y, and D, the variance of data segments during dynamic events is significantly higher than that in a static environment. Specifically, the locations of the data segments corresponding to dynamic events are almost identical across the three channels. By cumulatively summing the parameters of the sliding variance sequence at corresponding positions, the distinctions between dynamic events and static environments are further amplified. Therefore, this approach not only enables more precise separation of dynamic event data segments from the complete RSSI time series, but also enhances the system’s sensitivity to sensing dynamic events.

#### 3.3.4. Z-Score

In order to normalize the data from different dimensions to the quantitative scale and effectively reduce the uncertainty and error due to varying collection conditions and equipment differences, the sequence S is processed using z-score calculations to obtain the sequence Z. During real-time processing, the length of sequence S continuously changes, making fixed values impractical for computing average and variance parameters in z-score calculations. To address this issue, this study combines sliding window and long-term data processing methods for obtaining z-score average and variance parameters. By utilizing data segments strongly correlated with the currently processed data point, we compute the sliding average and sliding variance sequences for sequence S. The sliding window of sequence S is represented by $W_{S}$. Furthermore, the method to determine the sliding window length $L_{W_{S}}$ is as follows:

(12) $$L_{W_{S}}=\max\left\{L_{W_{X}},L_{W_{Y}},L_{W_{D}}\right\}$$

**Figure 4:**
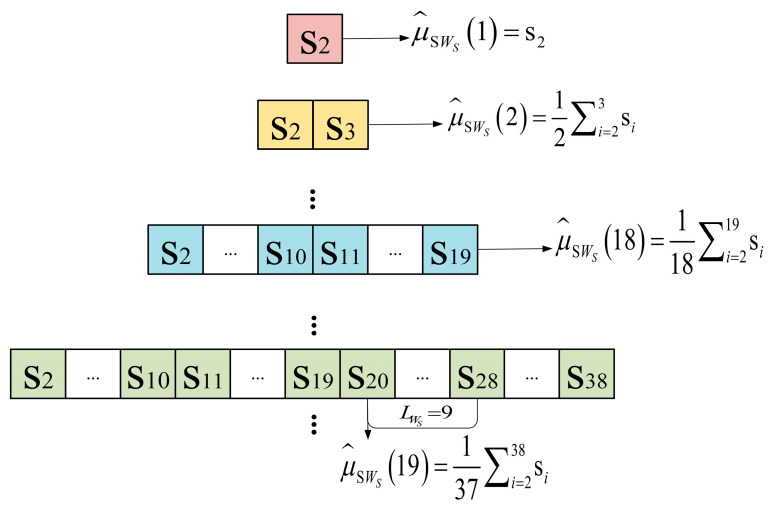
Schematic diagram of the sliding average sequence calculation of sequence S.

Using the average sliding window and long-term processing method, the sliding average sequence of the sequence S is calculated, as shown in Figure 4.

In sequence S, when the data point being processed by the average sliding window precedes the $2L_{W_{S}}+1$th data point, the parameter value of the sliding average sequence is computed using all preceding parameters in sequence S. Once the data point being processed reaches or exceeds the $2L_{W_{S}}+1$th data point, the long-term data analysis method is used. Centering on the data point being processed in the average sliding window, the method extends $2L_{W_{S}}$ data points both forward and backward to calculate the parameter value of the sliding average sequence. The sliding average sequence of sequence S is denoted by $\left\{{\hat{\mu }}_{SW_{S}}(1),{\hat{\mu }}_{SW_{S}}(2),{\hat{\mu }}_{SW_{S}}(3),...\right\}$. Taking Figure 4 as an example, with a sliding window length set to 9, the calculation of ${\hat{\mu }}_{SW_{S}}(19)$ involves 37 data points, including its own position and 18 data points extended both forward and backward.

In sequence S, the calculation method of the sliding average sequence parameter value ${\hat{\mu }}_{SW_{S}}(n)$ is as follows:

(13) $${\hat{\mu }}_{SW_{S}}(n)=\left\{\begin{array}{l} \frac{1}{n}\sum_{i=1}^{n}s_{i},0<n\leq 2L_{W_{S}}\\ \frac{1}{4L_{W_{S}}+1}\sum_{i=n-2L_{W_{S}}}^{n+2L_{W_{S}}}s_{i},n>2L_{W_{S}} \end{array}\right.$$

Furthermore, the sliding variance sequence of sequence S is denoted by $\left\{{\hat{\delta }}_{SW_{S}}^{2}(2),{\hat{\delta }}_{SW_{S}}^{2}(3),{\hat{\delta }}_{SW_{S}}^{2}(4),...\right\}$. The parameter value of the sliding variance sequence is represented by ${\hat{\delta }}_{SW_{S}}^{2}(n)$. Combined with the calculation result of the sliding average sequence parameter value ${\hat{\mu }}_{SW_{S}}(n)$, the calculation method of ${\hat{\delta }}_{SW_{S}}^{2}(n)$ is as follows:

(14) $${\hat{\delta }}_{SW_{S}}^{2}(n)=\left\{\begin{array}{l} \frac{1}{n}\sum_{i=1}^{n}{\left(s_{i}-{\hat{\mu }}_{SW_{S}}(n)\right)}^{2},1<n\leq 2L_{W_{s}}\\ \frac{1}{4L_{W_{S}}+1}\sum_{i=n-2L_{W_{S}}}^{n+2L_{W_{S}}}{\left(s_{i}-{\hat{\mu }}_{SW_{S}}(n)\right)}^{2},n>2L_{W_{S}} \end{array}\right.$$

In processing the average and variance sliding windows of sequence S, the non-overlapping sliding approach is used in conjunction with the long-term processing method to enhance the execution efficiency of RSSI-WSDE. By combining the parameter value of sequence S, the z-score sequence is obtained, denoted as sequence Z, which can be expanded as $\left\{z_{2},z_{3},z_{4},...\right\}$. The calculation method of data sample $z_{n}$ in sequence Z is as follows:

(15) $$z_{n}=\frac{s_{n}-{\hat{\mu }}_{SW_{S}}(n)}{\sqrt{{\hat{\delta }}_{SW_{S}}^{2}(n)}},n>1$$

Applying the z-score method during the data processing of the sequence S does not alter the form of the data distribution. This means that sequence Z still retains the distribution information of the data in sequence S.

#### 3.3.5. Threshold of Dynamic Event Occurrence

To sense the occurrence of dynamic events, it is essential to extract the data fluctuation pattern and distribution from the sensed time series data over the certain period. Therefore, we design and apply an adaptive event occurrence threshold for event sensing tailored to different scenarios, environments, and types of events. The threshold of dynamic event occurrence is denoted by $T_{h}$. In order to ensure the reasonableness and stability of the threshold settings, sequence Z needs to be further processed to achieve data cleaning and smoothing effects. The processed data in sequence Z not only meet the analysis requirement, but also retain the original characteristics and overall distribution trend of the data. After smoothing, the sequence Z of the collection unit device group $g_{i}$ is represented by set $\hat{Z_{g_{i}}}≜\{\hat{z_{2}},\hat{z_{3}},\ldots ,\hat{z_{n}}\}$. The data in the smoothed sequence are processed to one-decimal-place precision by rounding. Furthermore, the initial dynamic event occurrence threshold is represented by $T_{h_{0}}$, with its default value set as $\hat{z_{2}}$. Since $\hat{z_{2}}$ is the first data point in the smoothed sequence Z, it typically represents the static environment data collected by the collection unit device group. As new data points are continuously collected, they will be compared with $T_{h_{0}}$ to obtain the counting flags for three different situations.

$F_{l}$, $F_{n}$, and $F_{g}$ are used to denote the counting flags for below threshold, normal count, and above threshold, respectively. Their values depend on the data $\hat{z_{n}}$ from the smoothed sequence Z. Specifically, when $\hat{z_{n}}$ is within the range of $\left[0.8T_{h_{0}},T_{h_{0}}\right)$, $F_{l}$ is incremented; when $\hat{z_{n}}$ is equal to $T_{h_{0}}$, $F_{n}$ is incremented; and when $\hat{z_{n}}$ is within the range of $\left(T_{h_{0}},1.2T_{h_{0}}\right]$, $F_{g}$ is incremented. Therefore, we can obtain the following:

(16) $$F_{l}=F_{l}++,\;0.8T_{h_{0}}\leq \hat{z_{n}}<T_{h_{0}}$$

(17) $$F_{n}=F_{n}++,\;T_{h_{0}}=\hat{z_{n}}$$

(18) $$F_{g}=F_{g}++,\;T_{h_{0}}\leq \hat{z_{n}}<1.2T_{h_{0}}$$

Therefore, based on the conditions set forth, the adaptive dynamic event occurrence threshold $T_{h}$ can be obtained, denoted as follows:

(19) $$T_{h}=\left\{\begin{array}{l} 1.1T_{h_{0}},\;F_{g}>F_{n}\&\&F_{g}>F_{l}\\ T_{h_{0}},\;F_{n}\geq F_{g}\&\&F_{n}\geq F_{l}\\ 0.9T_{h_{0}},\;F_{l}\geq F_{g}\&\&F_{l}\geq F_{n} \end{array}\right.$$

The dynamic event occurrence threshold $T_{h}$ can be adaptively updated based on the changes in the quantitative relationship of the counting flag, so as to effectively sense dynamic event in various scenarios and environments.

#### 3.3.6. Dynamic Event Information Marking

Based on Equation (19), the threshold value $T_{h}$ of the dynamic event occurrence is obtained. Combined with sequence Z, it enables the marking of dynamic event occurrence.

During dynamic event wireless sensing, the sampling frequency of the device is represented by $f_{s}$. Additionally, the initial time of the wireless sensing task is recorded as $t_{0}$. Combined with $T_{h}$, the data index set representing the beginning time of the event is denoted by $I_{b}≜\left\{i_{b}^{1},i_{b}^{2},\ldots i_{b}^{k}\right\}$, and the data index set representing the ending time of the event is denoted by $I_{e}≜\left\{i_{e}^{1},i_{e}^{2},\ldots i_{e}^{k}\right\}$. By using the data index of the event beginning time, the specific time $ST_{k}$ of the kth event occurrence can be calculated. The method for calculating the specific time $ST_{k}$ of the kth event occurrence is as follows:

(20) $$ST_{k}=t_{0}+\frac{i_{b}^{k}}{f_{s}}$$

The list of specific times can be denoted as $\left\{ST_{1},ST_{2},ST_{3},...\right\}$. Furthermore, the duration of the kth event occurrence is represented by $DU_{k}$. The calculation method for the duration $DU_{k}$ of the kth event occurrence is as follows:

(21) $$DU_{k}=\frac{\left(i_{e}^{k}-i_{b}^{k}\right)}{f_{s}}$$

The list of durations can be denoted $\left\{DU_{1},DU_{2},DU_{3},...\right\}$. By combining the specific time and duration of the dynamic event, this study implements the marking function of dynamic event-related information within the waveform sequence Z, thereby achieving the purpose of dynamic event wireless sensing.

The algorithm flow of RSSI-WSDE is shown in Algorithm 1.
**Algorithm 1:** RSSI-WSDE**Input:** initial time *t_0_*, sampling frequency *f_s_*, and RSSI data in channels X and Y**Output:** specific time list ST_k__list, duration list DU_k__listn = 0Aligns the amount of RSSI data in both channels by padding zero at the end**while** (n < channel Y.size)Using channel Y as the reference channel, calculate the difference between the RSSI data of the two channels and populate it into Channel D.Calculate the sliding average sequence parameter values of three channelsCalculate the sliding variance sequence parameter values of three channelsCalculate the variance amplification sequence parameter values $s_{n}$Using long-term and sliding windows, calculate ${\hat{\mu }}_{SW_{S}}(n)$ and ${\hat{\delta }}_{SW_{S}}^{2}(n)$Calculate sequence Z parameter values $z_{n}$Calculate the threshold for dynamic event occurrence $T_{h}$Obtain the indices $i_{b}^{k}$ and $i_{e}^{k}$ of the event occurrence related dataCalculate $ST_{k}$ and $DU_{k}$ by combining *t_0_*, *f_s_*, $i_{b}^{k}$ and $i_{e}^{k}$ST_k__list.append($ST_{k}$), DU_k__list.append($DU_{k}$)n + 1**Return** ST_k__list and DU_k__list

First, the RSSI data amounts of channel X and channel Y are aligned by padding zeros at the end. Using channel Y as the reference channel, the difference between the RSSI data of the two channels is calculated and populated into channel D. Subsequently, the sliding average sequence and sliding variance sequence parameter values of all three channels are calculated. By accumulating the slider variance sequence parameter values from corresponding data points across the channels, the variance amplification sequence S is constructed. Furthermore, using sliding window techniques combined with the long-term data processing strategy, the sliding average sequence and the sliding variance sequence of sequence S are calculated. Moreover, by performing z-score calculation, sequence Z is obtained. Using sequence Z, the threshold is determined for sensing dynamic event occurrence and identifying the associated data indices. Ultimately, by utilizing the initial time, sampling frequency, and data indices for beginning and ending time of events, specific times and durations of dynamic events are determined and added to the corresponding list.

## 4. Experimental Testing

### 4.1. Experimental Setup

**Figure 5:**
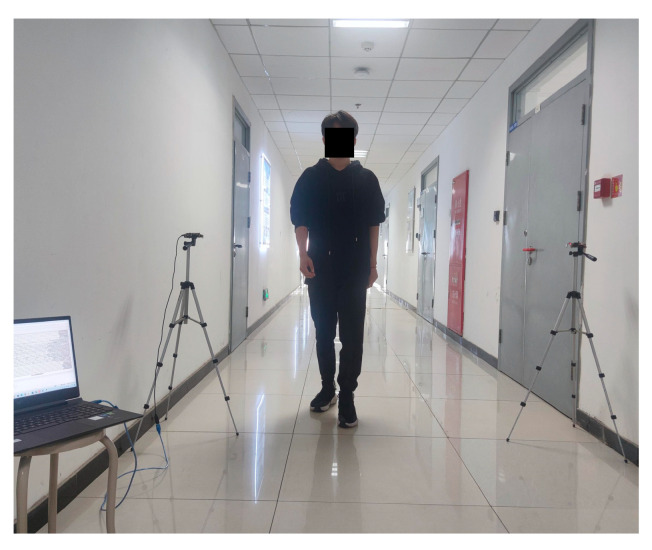
Indoor dynamic event data collection environment.

For dynamic event data collection, both indoor and outdoor environments are considered. The indoor data collection environment is shown in Figure 5.

The transmitter and the receiver units were placed on both sides of the corridor to collect the RSSI time series data of human activities occurring within the coverage area of the wireless sensing device. Under the condition of ensuring the normal operation of the devices, the distance between the transmitter and receiver unit was set to 1.5 m, with the placement height of 1.2 m.

During operation, the transmitter unit periodically sends data to the receiver unit, which utilizes the integrated RSSI module to calculate the forward channel RSSI. Then, the receiver unit transmits it back to the transmitter unit. Upon receiving the forward channel RSSI, the transmitter unit also utilizes the integrated RSSI module to calculate the backward channel RSSI.

Additionally, a host computer is connected to the transmitter device via a data transmission line and uses the serial port debugging assistant to view the RSSI dual-channel time series. In order to avoid the presence of the host computer affecting the experimental results, it is placed more than 1.5 m away from the transmitter unit. Moreover, during the device initialization phase, the connectivity of the channel between the transmitter and receiver unit can be verified using the serial port debugging assistant. The main parameters of serial port debugging assistant are configured as shown in Table 2.

The serial port parameters serve as the interface for serial communication between the host computer and external devices. During the experiment, it needs to be matched with the serial port that the driver operates. The baud rate measures the transmission speed of the serial data. In order to meet the requirements for high-speed data transmission, the experimental baud rate is set to 115,200 bps. The data bit is set to 8, allowing a complete byte to be transmitted in each data packet. Additionally, the start and stop bits are set to 1, ensuring sufficient intervals between data frames for the host computer to synchronize and parse the data correctly. The display mode of the reception area is set to hexadecimal, which facilitates quick data checking and error location. Specifically, hexadecimal display allows for more compact representation of transmitted data.

**Figure 6:**
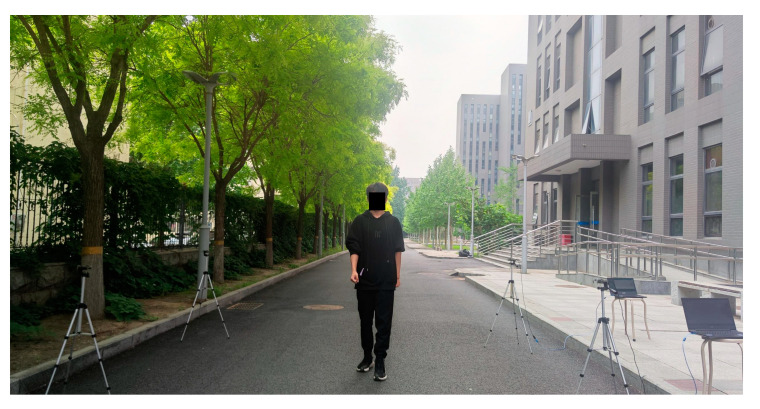
Outdoor dynamic event data collection environment.

The outdoor data collection environment is shown in Figure 6.

The transmitter unit and the receiver unit were placed on both sides of the road to collect the RSSI time series data of dynamic events such as walking, running, cycling, and driving. To ensure that all dynamic events can be performed within the experimental area, the distance between the transmitter and receiver unit was set to 4 m. Additionally, the placement heights of the devices were set at 1.2 m to ensure the normal collection of RSSI time series data of dynamic events. Similar to the indoor experiment, the RSSI for both the forward and backward channels is calculated using integrated RSSI module. Subsequently, the transmitter unit transmits the RSSI of dual-channel to the host computer via a data transmission line. The RSSI time series data are viewed on the host computer using the serial port debugging assistant. Specifically, for outdoor dynamic event data collection, two sets of collection unit device groups were used simultaneously to improve experimental efficiency. This approach helps to collect more dynamic event RSSI data in a short period of time.

### 4.2. Experimental Results of RSSI-WSDE

#### 4.2.1. Indoor Experimental Results

**Figure 7:**
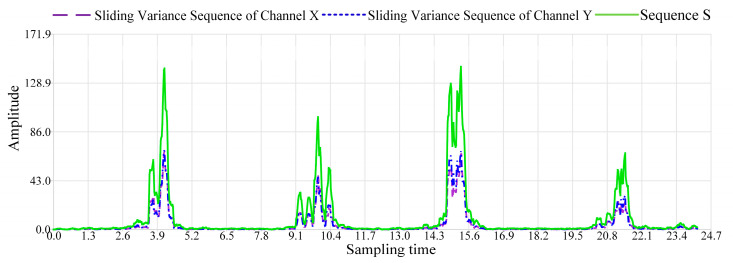
Variance amplification effect of sequence S.

In order to amplify the difference between the data of dynamic events and static environments in the original RSSI time series, the sequence S is obtained through variance amplification operation. The variance amplification effect of sequence S for an indoor back-and-forth walking dynamic event is shown in Figure 7.

Sequence S integrates the fluctuations in the sliding variance sequences of each channel and further amplifies the amplitude differences. Consequently, this effectively reduces the interference from static environment data on the determination of dynamic event occurrence. Moreover, it enhances the robustness and accuracy of the system in sensing dynamic events. Within sequence S, the waveform of static environment data exhibits a smooth trend, while the waveform of dynamic event data displays significant amplitude fluctuations.

**Figure 8:**
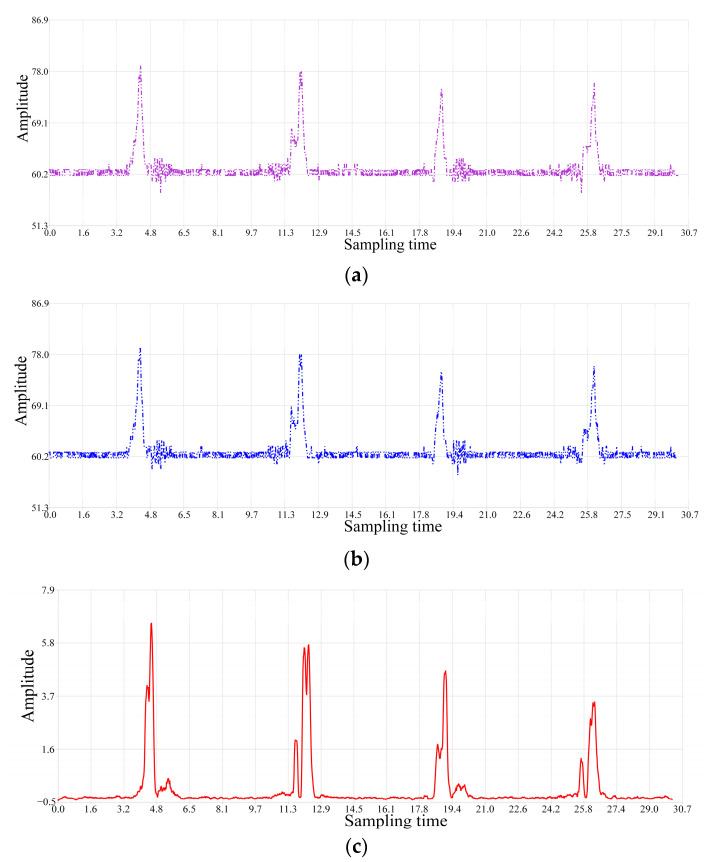
Comparison of the waveforms of sequence Z and the original acquired dual-channel data. (**a**) Original data waveform of channel X; (**b**) original data waveform of channel Y; (**c**) data waveform of sequence Z.

In order to enhance the comparability of data across different dimensions or amplitude fluctuation ranges, sequence Z is obtained through z-score calculation. This approach enables the extraction of potential dynamic event information from the RSSI time series data under a unified quantitative scale. A comparison of the waveform of the sequence Z for indoor back-and-forth walking dynamic events with the original data of the two channels at a sampling frequency of 100 Hz is shown in Figure 8.

When walking through the dynamic event wireless sensing area, the data amplitude significantly increases. In Figure 8, the horizontal axis represents the total time taken to collect two rounds of back-and-forth walking dynamic event RSSI time series data. Figure 8 shows four instances of amplitude increase, corresponding to four phases of two rounds of back-and-forth walking. The first and third amplitude increases indicate forward walking through the sensing area, while the second and fourth increases signify walking in the opposite direction through the sensing area. When walking reaches the line-of-sight path of the collection units, a peak amplitude occurs. Additionally, the starting and ending points of each walking phase are outside the effective range of the wireless sensing area. Therefore, once walking passes through the sensing area, the phenomenon of amplitude increase disappears. The system continues to collect RSSI data from the static environment.

Sequence Z retains the differences between dynamic events and static environmental data as reflected in sequence S. This effectively addresses the issue in the original RSSI time series of channels X and Y, where the specific times and durations of dynamic events cannot be accurately sensed due to the influence of static environment and noise. Furthermore, through z-score processing, the measurement scale of the waveform amplitudes is normalized, enabling the system to compare the sensing effects of different dynamic events.

**Figure 9:**
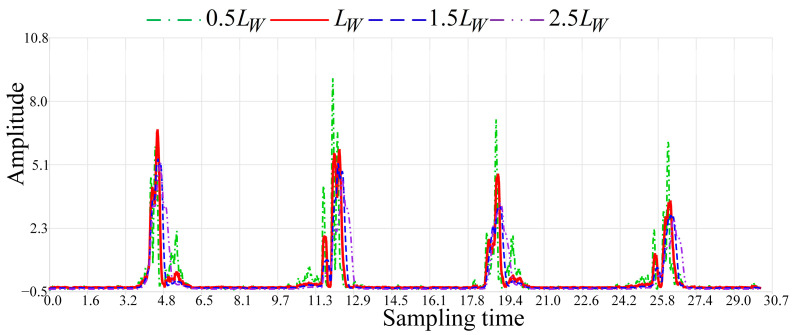
Comparison of sequence Z waveforms with different window lengths.

When the sliding window length $L_{W}$ varies, the waveforms of the sequence Z obtained by dynamic event sensing exhibit differences, as shown in Figure 9.

During dynamic event wireless sensing, the default length of the sliding window is set to 9 data points. Figure 9 compares the sensing results of indoor back-and-forth walking dynamic events under four different sliding window lengths at a 100 Hz sampling frequency. When the window length is set to $0.5L_{W}$, the smoothing effect on static environmental data is poor. There are many abnormal fluctuations in the waveform of sequence Z, reducing the accuracy of dynamic event wireless sensing. When the window lengths are set to $1.5L_{W}$ and $2.5L_{W}$, an over-smoothing phenomenon occurs for dynamic event data, reducing the ability to sense detailed information about dynamic events. When the window length is set to $L_{W}$, it can moderately smooth static environmental data while ensuring the effective sensing of dynamic event details. RSSI-WSDE is capable of matching the appropriate sliding window length according to different sampling frequencies.

**Figure 10:**
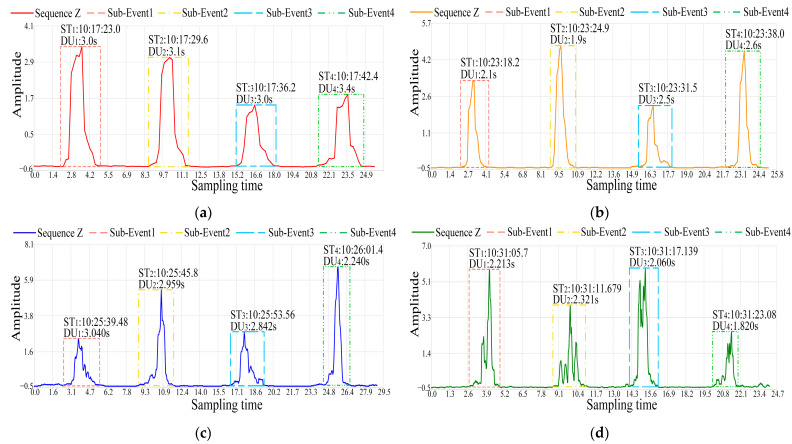
Sequence Z waveforms of the four sampling frequencies. (**a**) The sequence Z waveform with a sampling frequency of 10 Hz; (**b**) the sequence Z waveform with a sampling frequency of 20 Hz; (**c**) the sequence Z waveform with a sampling frequency of 50 Hz; (**d**) the sequence Z waveform with a sampling frequency of 100 Hz.

The RSSI time series data for indoor back-and-forth walking dynamic events were collected using devices with sampling frequencies of 10 Hz, 20 Hz, 50 Hz, and 100 Hz. The waveforms of sequence Z are shown in Figure 10.

Figure 10 shows the waveforms of sequence Z for back-and-forth walking dynamic events at four sampling frequencies of 10 Hz, 20 Hz, 50 Hz, and 100 Hz. The horizontal axis of the coordinate system represents the sampling time, while the vertical axis represents the amplitude of the sequence Z data. Sub-event 1 and sub-event 3 display the sequence Z waveforms of forward walking, while sub-event 2 and sub-event 4 display the sequence Z waveforms of backward walking.

For dynamic events occurring in different orders, single dynamic events are marked using bounding boxes with different colors and line styles. The positions of the four vertices of each bounding box are jointly determined by the beginning time, ending time, peak value, and trough value of the sequence Z amplitude changes during a single event occurrence. For the bottom-left vertex of the bounding box, its coordinates are determined with the beginning time of the single event as the abscissa and the trough value of the sequence Z amplitude changes as the ordinate. Additionally, above the bounding boxes of the dynamic event occurrences, the specific times $ST_{k}$ and durations $DU_{k}$ of each event are marked.

Furthermore, with the increase in the sampling frequency, the complexity of sequence Z is enhanced, enabling the system to sense more detailed information of dynamic events, manifested specifically in the clearer presentation of directional information. The sequence Z values of forward walking sub-events 1 and 3 exhibit strong similarity, with an overall waveform pattern showing an initial rise followed by a decline. In contrast, the sequence Z values of backward walking sub-events 2 and 4 demonstrate an overall pattern of an initial decline followed by an increase. However, due to the varying walking speeds of the experimenters, differences in the amplitudes and fluctuations of the waveforms have arisen, thereby reducing the clarity of directional information. As shown in Figure 10d, during sub-event 2, the experimenter walked at a relatively slow pace, causing significant oscillations in the sequence Z waveform data. Nonetheless, the overall trend of the waveform still remains similar to that of sub-event 4.

The wireless sensing results of indoor back-and-forth walking dynamic events by RSSI-WSDE at four different sampling frequencies $f_{s}$ are shown in Table 3.

Table 3 shows the performance of RSSI-WSDE for processing the dynamic events of back-and-forth walking in an indoor corridor environment under device conditions with sampling frequencies $f_{s}$ of 10 Hz, 20 Hz, 50 Hz, and 100 Hz. When $f_{s}$ is 10 Hz, the event occurrence threshold $T_{h}$ is set to −0.49, while in the other three sampling frequencies, $T_{h}$ is set to −0.39. The threshold $T_{h}$ can be fine-tuned based on the distribution characteristics of sequence Z to accurately determine the specific times $ST_{k}$ of each event. Additionally, compared with low sampling frequencies, high sampling frequencies demonstrate higher accuracy in sensing the durations of dynamic events. Specifically, at the sampling frequencies of 50 Hz and 100 Hz, the system achieves millisecond-level accuracy in sensing dynamic events.

Furthermore, the durations $DU_{k}$ of dynamic events at different sampling frequencies can reflect the speed changes of walking events. Taking the device with $f_{s}$ of 50 Hz as an example, the values of DU_1_, DU_2_, and DU_3_ are relatively long and close to each other, indicating relatively steady walking speeds in the first three events. In contrast to the durations of the first three walking events, the value of DU_4_ for the fourth walking event exhibits a significant decrease, reflecting the relatively fast walking speed on that occasion.

**Figure 11:**
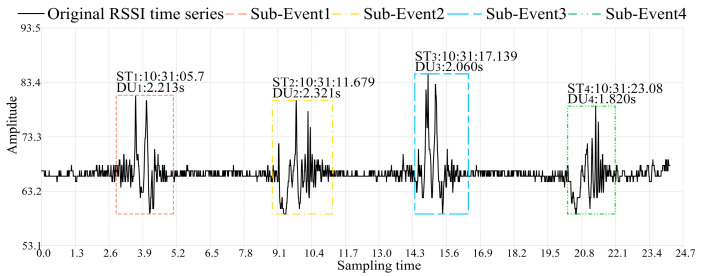
The final sensing effect on the original time sequence.

After obtaining the specific times and durations of the dynamic events, the detailed information is marked on the complete original RSSI time series to achieve the final effect of sensing the occurrence of dynamic events. Due to the processing time required by the algorithm, there is inevitably some delay in real-time sensing, as shown in Figure 11.

Figure 11 illustrates the sensing effect on the complete original RSSI time series of indoor back-and-forth walking dynamic events at a sampling frequency of 100 Hz. After RSSI-WSDE processing, the specific times and durations of dynamic events can be clearly marked in the original RSSI time series. Determining the specific time of event occurrence allows for precise localization of the event’s temporal point, thereby accomplishing the fundamental sensing task of dynamic events. Furthermore, the duration information allows for inference of differences in walking speeds. For instance, in Figure 11, DU_4_ is 1.820 s, indicating the fastest walking speed. DU_2_ is 2.321 s, indicating the slowest walking speed. The sensing result of RSSI-WSDE is closely aligned with the amplitude fluctuations in the original RSSI time series, thereby sensitively sensing the occurrence of dynamic events. The four vertices of the bounding box are jointly determined by the beginning time, ending time, peak, and trough of amplitude variations in a single event. Additionally, by setting an efficient threshold for dynamic event occurrence, abnormal amplitude fluctuations caused by static environments and noise can be filtered out to prevent the erroneous sensing of dynamic events.

#### 4.2.2. Outdoor Experimental Results

**Figure 12:**
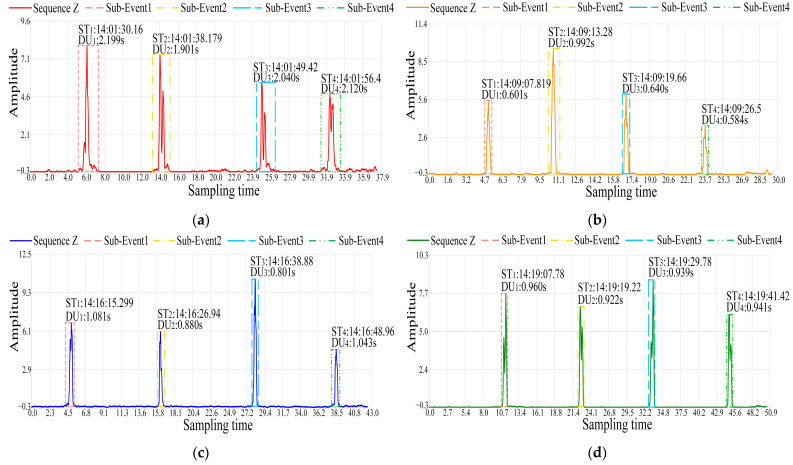
The waveform of sequence Z for various outdoor dynamic events collected at the sampling frequency of 100 Hz. (**a**) The sequence Z waveform of the walking event; (**b**) the sequence Z waveform of the running event; (**c**) the sequence Z waveform of the cycling event; (**d**) the sequence Z waveform of the driving event.

In outdoor environment, the RSSI time series data of dynamic events such as walking, running, cycling, and driving were collected. Each dynamic event followed a trajectory of two rounds of back-and-forth movements within the sensing area. The waveforms of sequence Z for various outdoor dynamic events collected using a device group with a sampling frequency of 100 Hz are shown in Figure 12.

In Figure 12, sub-event 1 and sub-event 3 represent forward movement for each type of dynamic event, while sub-event 2 and sub-event 4 indicate movement in the opposite direction. Due to the varying speed and volume of each outdoor dynamic event, there are differences in the clarity of the event occurrence detailed information. Compared with other dynamic events, walking is slower in execution and has a longer duration within the sensing area. Therefore, the direction information related to event occurrence is more pronounced in the walking event sequence Z. However, due to the smaller volume of walking events, it is not sufficient to accurately determine their directional information. As illustrated in Figure 12a, sub-events 2 and 4 exhibit pronounced directionality, whereas sub-events 1 and 3 display weaker directionality.

In dynamic events such as running and cycling, differences in execution speeds and volumes are relatively minimal. Furthermore, their impact durations within the sensing area are comparatively brief, leading to less pronounced directional information in the sequence Z of running and cycling events. However, the occurrence of the corresponding event can still be sensed.

The dynamic event of driving is characterized by high speed and large volume, resulting in relatively long event duration. Moreover, the directional information the of driving event is more pronounced. In the case of forward vehicle movement, the waveform of the driving dynamic event in sequence Z demonstrates a trend of initially high values followed by lower values. Conversely, for backward vehicle motion, the waveform shows the inverse trend.

The wireless sensing results of four outdoor dynamic event types $E_{t}$ at the sampling frequency of 100 Hz are shown in Table 4.

Table 4 shows the performance of RSSI-WSDE in sensing four outdoor dynamic event types $E_{t}$ for walking, running, cycling, and driving. In the same outdoor environment, RSSI-WSDE sets similar event occurrence thresholds $T_{h}$. When sensing the walking event, $T_{h}$ is set to −0.29. For sensing the other three dynamic event types, $T_{h}$ is set to −0.19. Specifically, RSSI-WSDE demonstrates robustness and adaptability in sensing different types of dynamic events. It can determine the specific times $ST_{k}$ of each dynamic event based on the threshold $T_{h}$.

Furthermore, analyzing the duration $DU_{k}$ can reflect the differences in the speeds and volumes among different events. The durations of the walking remain within the range of 1.9 s to 2.2 s, which is significantly longer than the other three types of events. These reflect the slower-speed characteristic of walking. Running events exhibit higher speeds compared to walking events, resulting in shorter durations. The speed of cycling is similar to that of running, but the volume of the cycling event is slightly larger than that of running, thus the duration is slightly longer. The volume of the driving event is significantly larger than the other three types of events, resulting in a longer duration of event occurrence.

## 5. Conclusions

In this study, we propose a novel wireless sensing method for dynamic events: RSSI-WSDE. In indoor corridor and outdoor urban road scenarios, RSSI-WSDE combines statistics methods with sliding window processing to achieve the wireless sensing of dynamic events. RSSI-WSDE calculates the sliding average sequence, sliding variance sequence, and variance amplification sequence to enhance the difference between dynamic events and static environment RSSI data. Subsequently, using the z-score, data are normalized to a unified quantitative scale, resulting in sequence Z. Moreover, adaptive event occurrence thresholds are set to sensing the dynamic events, and mark their specific times and durations on the original RSSI time series.

Specifically, by using RSSI-WSDE, it is possible to separate dynamic events from the complete RSSI time series that includes static environment data, thereby accurately sensing the occurrence of dynamic events. Furthermore, RSSI-WSDE provides a new perspective for building smart cities. By deploying it in auxiliary devices for dynamic event sensing, the real-time sensing of dynamic events at various traffic nodes is enabled. This enhances the effectiveness of public security departments in criminal investigation operations. Additionally, it supplements the capabilities for rapid monitoring and tracking of suspects or vehicles in a dark environment.

In future work, we will focus on studying the directionality of dynamic event occurrence. This will enable the algorithm to sense the directionality of different dynamic events at various sampling frequencies. Furthermore, it is crucial to integrate the characteristic information extracted from this study, such as the durations of dynamic event occurrences and changes in waveform data amplitude. This integration will facilitate the design of dynamic event classification algorithms suitable for indoor and outdoor scenarios, enhancing the granularity of dynamic event sensing. Additionally, based on the directionality and classification results of dynamic events, it is practicable to further design a novel method for reconstructing the motion trajectories of target objects. This approach can enhance the diversity of functionalities and response capabilities of the dynamic event sensing system.

## Figures and Tables

**Table 1:** List of main notations.

Notation	Description
G	The set of collection unit device groups
$g_{i}$	The *i*th collection unit device group
$W_{N},N\in \left[X,Y,D\right]$	The sliding window
$L_{W_{N}},N\in \left[X,Y,D\right]$	The sliding window length
$\mu_{NW_{N}}(n),N\in \left[X,Y,D\right]$	The parameter of the sliding average sequence
$\sigma_{NW_{N}}^{2}(n),N\in \left[X,Y,D\right]$	The parameter of the sliding variance sequence
$cov_{W_{D}}(X,Y)$	The covariance of the data processed through sliding window $W_{D}$
$s_{n}$	The data sample in sequence S
${\hat{\mu }}_{SW_{S}}(n)$	The parameter of the sliding average sequence in sequence S
${\hat{\delta }}_{SW_{S}}^{2}(n)$	The parameter of the sliding variance sequence in sequence S
$\hat{z_{n}}$	The smoothed data sample in sequence Z
$T_{h}$	The dynamic event occurrence threshold
$T_{h_{0}}$	The initial dynamic event occurrence threshold
$F_{N},N\in \left[l,n,g\right]$	The counting flag
$f_{s}$	The sampling frequency of the device
$t_{0}$	The initial time of the wireless sensing task
$i_{N}^{k},N\in \left[b,e\right]$	The data index representingthe beginning time and the ending time of the *k*th event
$ST_{k}$	The specific time of the *k*th event occurrence
$DU_{k}$	The duration of the *k*th event occurrence

**Table 2:** Main parameters of serial port debugging assistant.

Parameter	Value
serial port	driver matching port
baud rate	115,200
start bit	1
data bit	8
stop bit	1
acceptance area display format	hexadecimal number

**Table 3:** Comparison of wireless sensing results of indoor back-and-forth walking events.

f_s_	T_h_	ST_1_	DU_1_	ST_2_	DU_2_	ST_3_	DU_3_	ST_4_	DU_4_
10 Hz	−0.49	10:17:23.0	3.0 s	10:17:29.6	3.1 s	10:17:36.2	3.0 s	10:17:42.4	3.4 s
20 Hz	−0.39	10:23:18.2	2.1 s	10:23:24.9	1.9 s	10:23:31.5	2.5 s	10:23:18.2	2.6 s
50 Hz	−0.39	10:25:39.48	3.040 s	10:25:45.8	2.959 s	10:25:53.36	2.842 s	10:26:01.4	2.240 s
100 Hz	−0.39	10:31:05.7	2.213 s	10:31:11.679	2.321 s	10:31:17.139	2.060 s	10:31:23.08	1.820 s

**Table 4:** Comparison of wireless sensing results for four outdoor dynamic events.

Et	T_h_	ST_1_	DU_1_	ST_2_	DU_2_	ST_3_	DU_3_	ST_4_	DU_4_
walking	−0.29	14:01:30.16	2.199 s	14:01:38.179	1.901 s	14:01:49.42	2.040 s	14:01:56.4	2.120 s
running	−0.19	14:09:07.819	0.601 s	14:09:13.28	0.992 s	14:09:19.66	0.640 s	14:09:26.5	0.584 s
cycling	−0.19	14:16:15.299	1.081 s	14:16:26.94	0.880 s	14:16:38.88	0.801 s	14:16:48.96	1.043 s
driving	−0.19	14:19:07.78	0.960 s	14:19:19.22	0.922 s	14:19:29.78	0.939 s	14:19:41.42	0.941 s

## Data Availability

The data are not publicly available as the project is not fully complete, so the data cannot be disclosed for the time being.
